# High-fidelity telesimulation to teach lactation skills to primary care residents, a cluster randomized educational trial

**DOI:** 10.1186/s12909-025-08118-2

**Published:** 2025-11-10

**Authors:** Adrienne Hoyt-Austin, Anna Sadovnikova, Sean Muñoz, Lee T Donohue, Daniel J Tancredi, Laura R Kair

**Affiliations:** 1 Division of General Pediatrics, Department of Pediatrics, University of California at Davis, 2516 Stockton Blvd, Sacramento, CA 95817 USA; 2 Division of Breastfeeding and Lactation Medicine, University of Rochester, 601 Elmwood Ave, Rochester, NY USA; 3 Division of Neonatology, Department of Pediatrics, University of California at Davis, 2516 Stockton Blvd, Sacramento, CA 95817 USA

**Keywords:** Breastfeeding education, Graduate medical education, Simulation, Telemedicine, Telesimulation

## Abstract

**Background:**

Most primary care physicians lack the clinical knowledge and skills to manage common breastfeeding problems. High-fidelity simulation with standardized patients in videoconferencing or telesimulation increases medical student confidence and competence in clinical lactation skills; yet it is not known if telesimulation at the graduate medical education level results in the translation of lactation skills in medical trainees.

**Objective:**

To assess the effect of a telesimulation versus videoconferencing didactics on clinical lactation practice patterns in resident trainees.

**Methods:**

In this randomized trial (NCT04519216) pediatric and family medicine residents were randomized to receive one breastfeeding education experience via either [1] traditional didactics through videoconferencing (control) or [2] telesimulation (intervention). The primary outcome was the change in composite standardized clinical lactation practice pattern scores from enrollment to 3 months. Practice patterns and clinical lactation self-efficacy were also collected and reported at post-test, 2-weeks, and 3 months. We conducted a difference-in-differences analysis of mean change scores and a sensitivity analysis was also completed to account for any missing data. Internal validity of the clinical lactation self-efficacy and standardized practice patterns survey batteries were assessed using Cronbach’s alpha.

**Results:**

62 residents received breastfeeding education; of which 51 participated in the study at any time. The mean change in composite practice pattern scores from baseline to 3 months increased in the intervention group to 1.3 (*±* 1.1) with the control group steady with a score of 0.3 (*±* 1.0). This difference was not significant as the effect size (per-protocol) was − 1.0 (-2.0, 0.1). Mean change scores from enrollment to 3 months showed an increase in clinical lactation self-efficacy for both groups with a gain of 1.6 (*±* 0.9) for control and 1.3 (*±* 0.8) for intervention; however, the effect size (per-protocol) of 0.3 (-0.5, 1.1) showed no significant difference between groups.

**Conclusions:**

Teaching breastfeeding medicine concepts and skills for graduate medical education level learners is important. We no significant difference in practice patterns between residents taught breastfeeding medicine concepts through videoconferencing didactics versus telesimulation.

## Introduction

Establishing and supporting exclusive breastfeeding has the potential to prevent over 3,000 maternal and infant deaths per year in the United States (US) and over $100 billion in healthcare costs [1, 2]. Thus, it is crucial for primary care physicians to have confidence and skills in providing evidence-based information to parents about breastfeeding and troubleshoot issues as they arise. Following birth hospitalization discharge, newborns are recommended to receive a health evaluation, commonly referred to as the newborn weight check, within 24–48 h for most deliveries [3]. At the newborn weight check, difficulties with breastfeeding including painful latch, engorgement, and concerns about delay in secretory activation (milk coming in) can be a common part of primary care [4]. Despite this critical role primary care physicians have in promoting breastfeeding, many feel unprepared to counsel families to facilitate breastfeeding due to a lack of preparation and knowledge about breastfeeding [5], and thus lends for the increased necessity for a more optimized training experience for primary care physicians.

A national survey of pediatric and family medicine program directors indicated that on average their residents obtain only 8–9 h of breastfeeding education in the entirety of a 3-year residency program [6, 7]. The COVID-19 pandemic dramatically changed medical education and removed many in-person teaching opportunities for our future physicians [8]. As much of medical education has transitioned to online methods, one way to continue to engage learners is through videoconferencing in a telesimulation environment with standardized patients. High-fidelity simulation with standardized patients in a videoconference (telesimulation) increases medical student and midwifery student’s confidence and competence in clinical lactation skills [9, 10]. Standardized patients can simulate common medical problems, such as breastfeeding difficulties, when using a high-fidelity simulator [11]. Technology-enhanced simulation in medical training has been applied to many domains of medical education and is associated with significant positive change in knowledge, skills, and behaviors in clinicians [12]. However, utilization of high-fidelity simulation for teaching core concepts in breastfeeding medicine via teleconferencing with standardized patients for primary care residents, to our knowledge, has not been described.

The purpose of this study is to establish the utility of high-fidelity telesimulation in teaching core concepts in breastfeeding medicine in optimizing the level of preparedness for pediatric and family medicine physician residents to provide timely and skilled lactation support.

## Methods

### Setting and participants

This study was conducted at a large academic hospital in Northern California that supports over 22 ACGME accredited residency training programs and 82 ACGME and university sponsored (non-ACGME) fellowship programs. Participants in this study were residents in the pediatric and family medicine residency programs completing a 4-week inpatient well newborn rotation at the main university hospital. The clinical team in the well newborn rotation was comprised of 1 attending, 1 pediatric senior resident (PGY-3), and two intern residents (1 each from family medicine and pediatrics). The hospital admits 1500–2000 infants for birth hospitalization per year both in the newborn nursery and the neonatal intensive care unit.

### Pilot study design

In this pilot cluster randomized trial, we studied two modes of delivery of clinical lactation education to physician residents and how that education impacted their self-efficacy and practice patterns. As this study was conducted during the height of the COVID-19 pandemic, both intervention and control groups received education through videoconferencing. Intervention groups completed a clinical lactation standardized patient encounter (herein telesimulation) and control groups completed a didactic educational session via Zoom (Version 5.0). Residents were cluster randomized by 4-week well newborn rotation block from July 2020 – June 2021. All residents on the well newborn rotation completed the education as described. No residents were able to cross over into another educational session as each rotation block was pre-set by the residency assigned schedule. All residents were invited to participate in the educational study; however, enrollment was optional. Participants received remuneration after completion of the final survey with a $5 gift card.

### Logistics of the telesimulation and videoconferencing didactics

The education included asynchronous pre-work online within Canvas Learning Management System (Canvas) to prepare the residents for the videoconference based educational session (telesimulation for intervention groups, videoconferencing didactics for control). The asynchronous pre-work included completion of one unfolding case scenario about maternal breast engorgement in the setting of delayed lactogenesis, previously studied in medical students [9]. All residents in this study answered five multiple-choice questions that included lactation physiology, physical assessment, clinical decision making, and management of clinical lactation concerns.

**Fig. 1:**
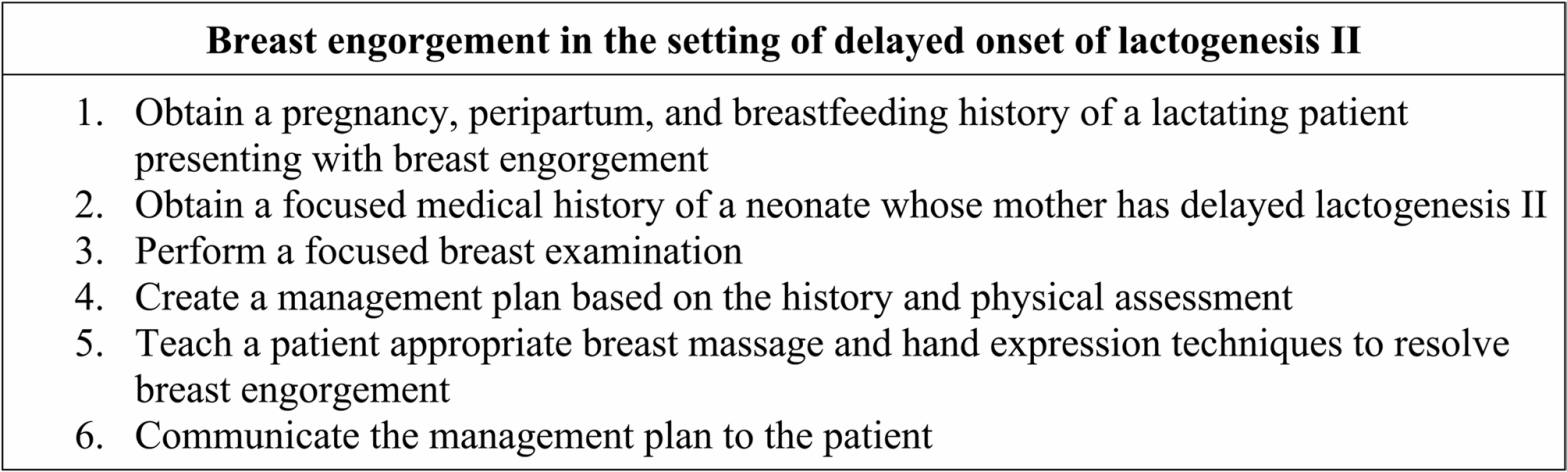
Learning objectives for the unfolding clinical lactation case scenarios completed by all pediatric and family medicine residents prior to videoconferencing didactics (control) or telemedicine standardized patient encounter (intervention)

After completion of the pre-work, intervention group participants were able to self-schedule the telesimulation and complete it asynchronously within Canvas. Control group participants were scheduled to a small group videoconferencing didactics session. Those randomized to the intervention completed a 30-minute telesimulation standardized patient encounter which included 20 min of videoconferencing with the standardized patient followed by 10 min of oral feedback from the standardized patient. Control group participants underwent a traditional didactic session in small group format using Zoom videoconferencing. The didactics were led by a general pediatrician (A.H.A) and included the newborn nursery team (senior resident, two first-year residents, and 0–3 medical students). Prior to study rollout, the didactic videoconferencing encounter with A.H.A underwent several rounds of internal and external review to ensure consistency. A mock didactic session by A.H.A was also assessed by the site university’s education department consistency in the delivery of the education. The case and learning objectives in the intervention and control groups was the same (see Fig. 1).

### The telesimulation encounter design and implementation

Standardized patients in the telesimulation encounters were lactation consultants or certified lactation counselors who had undergone extensive training in the case. They also had training in provision of targeted clinical lactation feedback immediately following the encounter using a validated formative and summative assessment instrument [10]. The standardized patient training protocol used for this study has been described in prior work by Anderson and colleagues where their group validated telesimulation as a method of teaching clinical lactation skills to medical students [9, 13]. The standardized patient encounter telesimulation sessions were conducted through LiquidGoldConcept™ via an institutional license for use in this study. Standardized patients wore a high-fidelity lactation simulation model in case of need for telemedicine examination or questioning by the physician trainees [11]. 

The case covered expected complications that can arise after birth hospitalization discharge including breast engorgement, delay in secretory activation, and neonatal hyperbilirubinemia. The didactics were led by a general pediatrician (A.H.A) and included the newborn nursery team (senior resident, two first-year residents, and 0–3 medical students).

### Outcomes and measures

At study enrollment, participants completed a deidentified enrollment survey with demographic information and pretest data regarding participant clinical lactation self-efficacy and practice patterns. Follow-up surveys were completed immediately post-test, at two weeks, and then three months following the educational session.

**Fig. 2:**
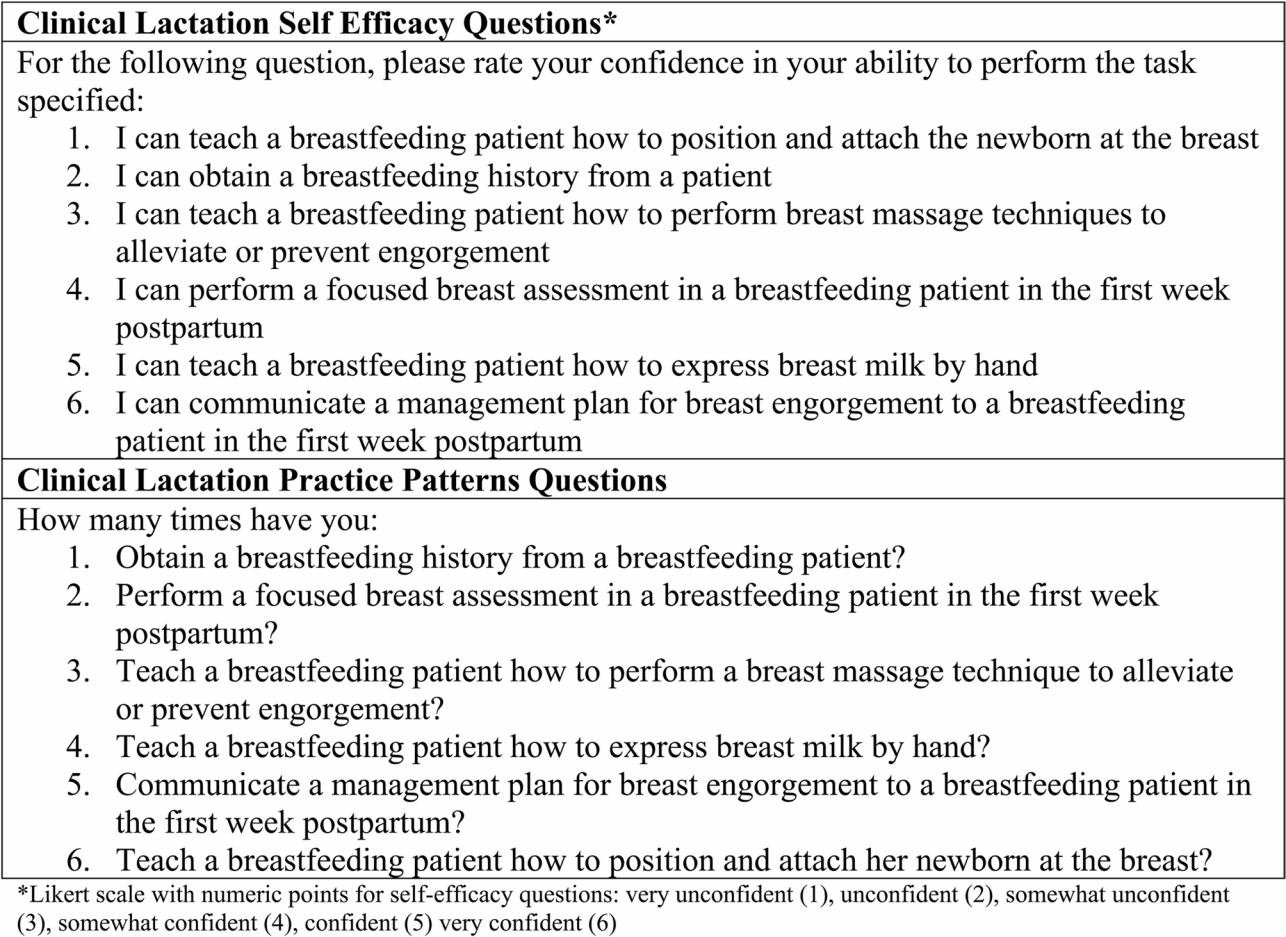
Clinical lactation self-efficacy and practice pattern instrument

The clinical lactation self-efficacy and practice patterns questions are listed in Fig. 2. The self-efficacy questions asked six questions about self-confidence in obtaining a breastfeeding history, completing a breast examination of a lactating patient, assisting with infant latch, and providing clinical lactation advice for common breastfeeding concerns. Participants rated their confidence on a 6-point Likert scale from “very unconfident” corresponding to a score of 1 to “very confident” corresponding to a score of 6. The clinical lactation practice patterns component of the survey asked six questions about the frequency in performing each of 6 skills including obtaining a breastfeeding history, teaching latch, and communication and provision of clinical advice for patients experiencing common breastfeeding concerns. Participants were asked to self-report the number of times they recalled ever completing a clinical lactation skill (practice pattern). Participants could indicate anywhere from 0 to *≥* 10 prior experiences of providing a particular practice pattern.

The survey tool was developed from the self-confidence and practice patterns survey by Feldman-Winter and colleagues [14]. Immediately following the educational session, regardless of intervention or control group, participants were surveyed regarding their confidence following the education. Surveys were distributed using the Qualtrics Experience Management Platform software and could be completed on a smart phone/tablet device or computer. Clinical lactation self-efficacy and practice patterns were surveyed again at two weeks and three months post-education. Non-responders were sent follow-up surveys up to three times.

Internal validity of the clinical lactation self-efficacy and standardized practice patterns survey batteries were assessed using Cronbach’s alpha. Because the means and standard deviations of the individual practice pattern skill frequency items varied, we applied a z-score transformation to these scale frequency items at all time points using the baseline means and standard deviations for each activity. Each z-score transformed skill frequency (practice pattern) was Winsorized to lie within the range of −3 to 3 to lessen the influence of outliers. Self-efficacy and composite standardized practice pattern scores were computed then as the mean of the non-missing items when no more than half of items were missing and otherwise were set to missing.

Additional analysis conducted includes a difference-in-differences analysis (t-test) of mean change scores in clinical lactation self-efficacy and composite standardized practice pattern scores by group. A sensitivity analysis was also completed to account for the missing data. In the sensitivity analysis, values for the change score for missing data were deemed zero since the results were not significant. All analyses were conducted using SAS OnDemand for Academics [15]. 

The primary outcome for this study was the change in practice patterns from enrollment to 3 months among residents randomized to the intervention and control groups. A sample size calculation was performed based on preliminary data collected at the same site [16]. In this study, however, we used an alternative measure of change in practice patterns, where a change of approximately one event was approximately one standard deviation. We determined that a sample size of 20 participants per group would allow us to achieve 87% power to detect a one standard deviation change in practice pattern and 99% power to detect a two standard deviation change practice patterns. This study was reviewed by the study site institutional review board and was determined to be exempt. This study was registered with ClinicalTrials.gov on August 12, 2020, with study identification number NCT04519216.

## Results

### Study participants

During the study period, 62 pediatric and family medicine physician residents received breastfeeding education during their newborn nursery rotation. All 62 residents completed the education and were invited to participate in the study and during the study period 51 residents participated (see Table 1). Most participants were pediatric residents, were female, and few (*n* = 3) participants reported prior personal breastfeeding experience. Thirteen had no prior clinical lactation training; of those with prior training, most reported receipt of lectures or didactics in medical training (55%).

**Table 1:** Participant demographics by group

Characteristic	All participants *N* = 51	Control *N* = 29	Intervention *N* = 22
Gender*			
Female	34	20	14
Male	9	5	4
Non-binary	1	1	0
Age*	28.9 *±* 3.4	28.6 *±* 3.55	29.1 *±* 3.26
Race/Ethnicity* - participants could select multiple			
Asian	14	9	5
Caucasian	24	14	10
Other	4	2	2
American Indian/Alaska Native	1	1	0
Hispanic/Latinx	5	4	1
Black	0	0	0
Participants with children	2	2	0
Breastfeeding experience			
None	41	23	18
Personal experience with breastfeeding	1	1	0
Personal experience supporting a breastfeeding partner	1	1	0
Other personal experience	1	1	0
Residency program*			
Pediatrics	26	16	10
Family Medicine	18	10	8
Prior clinical lactation training*			
No prior training	13	5	8
Lectures/didactics in medical school or residency	28	19	10
Simulation-based training in med school or residency	1	0	1
Supervised care of breastfeeding patients in med school or residency	4	2	2
IABLE or ABM training**	5	4	2
Other (HRIA, research)	4	2	2

*Data from 7 participants missing

**ABM Academy of Breastfeeding Medicine, *IABLE* Institute for Advancement of Lactation Education

### Consistency testing

All participants were asked about their clinical lactation practice patterns and self-efficacy at enrollment, 2 weeks and 3 months post education (Fig. 2). We conducted internal consistency testing of the questionnaires using Cronbach alpha. The Cronbach alpha of clinical lactation confidence scores and standardized composite practice pattern scores at enrollment was 0.87 and 0.83 respectively.

### Participant clinical lactation self-efficacy

At pre-test, the mean clinical lactation self-efficacy scores were 2.6 (*±* 0.8), between “Unconfident” and “Somewhat unconfident”. Separated by group, mean clinical lactation self-efficacy scores at pre-test were 2.4 (*±* 0.5) for intervention group participants and 2.7 (*±* 1) for control group participants (Table 2). All participants, regardless of study arm, had a positive change in mean change scores gain in clinical lactation self-efficacy scores following the education, with total mean scores immediately post-education of 4 (*±* 0.9), 4.1 (*±* 0.8) at two weeks, and 4.1 (*±* 0.6) at 3-months post education, correlating to “Somewhat confident” (Table 2).

**Table 2:** Clinical lactation self-efficacy and practice patterns at follow up time points

Variable	Mean score/practice patterns (SD)	Intervention *N* = 22 mean (SD)	Control *N* = 29 mean (SD)
Self-efficacy mean score			
Pre-test	2.6 (0.8)	2.4 (0.5) *n* = 18	2.7 (1) *n* = 26
Post test	4 (0.9)	3.7 (0.9) *n* = 16	4.2 (0.9) *n* = 21
2 week	4.1 (0.8)	3.8 (0.7) *n* = 10	4.3 (0.8) *n* = 17
3 month	4.1 (0.6)	3.7 (0.4) *n* = 9	4.3 (0.7) *n* = 14
Composite standardized practice pattern* mean score			
Pre-test	0 (0.6)	−0.3 (0.3) *n* = 17	0.1 (0.8) *n* = 23
2 week	1.1 (1)	0.7 (1.1) *n* = 8	1.4 (0.9) *n* = 14
3 month	0.6 (1.1)	0.9 (1.1) *n* = 9	0.4 (1.2) *n* = 14

*Composite standardized practice patterns are z-score transformed skill frequencies (practice pattern) that were Winsorized to lie within the range of -3 to 3 to lessen the influence of outliers

All participants had an increase in their clinical lactation self-efficacy scores; however, there were no significant differences in the effect size for mean-change scores between intervention and control arms at any time point (Table 3). Because of a high dropout rate of participants over time (see Tables 2 and 3 for the n for each item), we conducted an intention-to-treat sensitivity analysis. We found no significant differences in effect sizes between intervention and control groups at any time (Table 3).

**Table 3:** Mean change scores in clinical lactation self-efficacy and composite standardized practice patterns (from pre-test) with effect sizes per protocol and intention-to-treat sensitivity analysis

	Control *n* = 29, mean (SD)	Intervention *n* = 22, mean (SD)	Effect size per protocol (95% CI)	Effect size intention to treat (95% CI)
Self-efficacy mean change score				
Post test	1.5 (1.0) *n* = 21	1.2 (0.6) *n* = 13	0.4 (−0.2, 1.0)	0.4 (−0.1, 1.0)
2 week	1.5 (1.1) *n* = 16	1.3 (0.9) *n* = 7	0.2 (−0.8, 1.2)	0.4 (−0.1, 1.0)
3 month	1.6 (0.9) *n* = 14	1.3 (0.8) *n* = 8	0.3 (−0.5, 1.1)	0.3 (−0.2, 0.8)
Composite standardized practice patterns* mean change score				
2 week	1.1 (1.1) *n* = 12	0.8 (1.2) *n* = 6	0.4 (−0.8, 1.5)	0.3 (−0.2, 0.7)
3 month	0.3 (1.0) *n* = 13	1.3 (1.1) *n* = 7	−1.0 (−2.0, 0.1)	−0.3 (−0.7, 0.2)

*Composite standardized practice patterns are z-score transformed skill frequencies (practice pattern) that were Winsorized to lie within the range of -3 to 3 to lessen the influence of outliers

### Participant practice patterns

At pre-test, the composite standardized practice pattern scores for all participants were 0 (*±* 0.6) for all participants, with intervention group participants at −0.3 (*±* 0.3) and control group participants at 0.1 (*±* 0.8). All participants, regardless of study arm, had an increase in practice patterns at 2-weeks post-education with composite standardized practice patterns increasing to 1.1 (*±* 1). However, there were no significant differences in the effect size between intervention and control groups, in both the per protocol and intention-to-treat sensitivity analysis (Table 3). At 3 months post-education, the mean composite standardized practice pattern score for all participants was 0.6 (*±* 1.1), with intervention group participants at 0.9 (*±* 1.1) and control group participants at 0.4 (*±* 1.2). At 3 months, the mean change score for composite standardized practice patterns (primary outcome) for the control group of 0.3 (*±* 1.0) and intervention group 1.3 (*±* 1.1) with no significant difference in the effect size in either the per protocol nor intention to treat analysis (Table 3).

## Discussion

This study reports on the first randomized trial in graduate-level physician trainees assessing a telelactation virtual standardized patient encounter versus videoconferencing traditional didactics via case-based presentation. We demonstrated that high-fidelity telesimulation can be used to teach educational concepts in breastfeeding medicine to primary care resident physicians. Both forms of education, telesimulation and videoconferencing didactics, were associated with positive sustained change in clinical lactation self-efficacy, without significant differences by educational method.

Studying the impact of medical education and when to assess outcomes can be challenging. In this study, we used self-assessment surveys to measure clinical lactation self-efficacy and practice patterns prior to receipt of the education, 2 weeks, and 3 months post education. Self-assessment in medical education, while widely used [17], does have limitations including social desirability bias and recall bias. We found variability in self-reported clinical lactation practice patterns at the 2-week versus 3 month follow up which we attribute to the availability to practice recently learned skills on rotations following the 4-week newborn nursery rotation. In this study, residents were able to immediately apply their skills while on the newborn nursery rotation. However, following the rotation, residents could be learning in any environment in the hospital such as the emergency department, inpatient medical wards, or intensive care service (to name a few) which would have limited opportunities to apply their clinical lactation skills. Future study should consider capturing patient centered outcomes to assess the impact of breastfeeding education for resident physicians, such as any change in breastfeeding rates during birth hospitalization.

Even though COVID-19 pandemic is over, telemedicine and has dramatically changed the medical education and patient care landscape [18]. Structured virtual telemedicine standardized patient encounters continue to be used in the undergraduate and graduate medical education setting to prepare medical trainees for patient care encounters [19–22]. Teaching clinical lactation through telelactation cases can be used to teach medical trainees and should be incorporated into primary care medical curricula. Notably, telesimulation is a cost-effective approach to distribution of the standardized patient encounter as it reduces total costs that can incur with in-person simulation, such as supply costs and clinical station rental [23]. 

Strengths of this study include its novel use of videoconferencing methods to provide clinical lactation training to primary care physician trainees during the height of the pandemic. While we did not pilot test the survey questions for external validity, we did confirm internal validity of survey questions with Cronbach’s alpha. External validity testing would be recommended when implementing educational strategies in varied graduate level training. Lastly, our study is also strengthened by assessing multiple levels of educational outcomes, including engagement, knowledge and confidence, and behavior change. We used asynchronous prework for all learners to improve engagement and preparation for the educational session, either telesimulation or didactics. Best practices in medical education are to include multiple levels of learner assessment, as recommended by the New World Kirkpatrick Model [24, 25]. 

While there was high engagement in the educational sessions in both intervention and control groups, the study was weakened by a lack of sustained participant engagement over time which impacted our ability to assess differences by treatment arm. We were able to address this weakness by including a sensitivity analysis and did not find significant differences in practice patterns nor self-efficacy by educational method. Lack of significance could have reflected a variety of variables, including inadequate sample size and differential opportunities to apply clinical lactation skills in rotations outside of the newborn nursery. Lastly, follow up of participants was limited by in-person contact limitations that were placed by the study site office of research during the height of the pandemic. The limitations of this study could impact the generalizability of this work to other sites or for other learners, especially because of the constraints of conducting this study at the height of the pandemic.

## Conclusion

In conclusion, provision of clinical lactation education to primary care trainees can be achieved through telelactation standardized patients as well as through traditional didactic educational sessions. Future study is needed to adequately determine the difference between telelactation standardized patients and usual medical education.

## Data Availability

The datasets used and/or analyzed during the current study are available from the corresponding author on reasonable request.
